# Estimated Prevalence of Resident-to-Resident Aggression in Assisted Living

**DOI:** 10.1001/jamanetworkopen.2024.9668

**Published:** 2024-05-03

**Authors:** Karl Pillemer, Jeanne A. Teresi, Mildred Ramirez, Joseph Eimicke, Stephanie Silver, Gabriel Boratgis, Rhoda Meador, Leslie Schultz, Jian Kong, Katja Ocepek-Welikson, E-Shien Chang, Mark S. Lachs

**Affiliations:** 1 College of Human Ecology, Cornell University, Ithaca, New York; 2 Division of Geriatrics and Palliative Medicine, Weill Cornell Medical College, New York, New York; 3 Columbia University Stroud Center at New York State Psychiatric Institute, New York; 4 Department of Medicine, Division of General Medicine, Columbia University Irving Medical Center, New York, New York; 5 Bronfenbrenner Center for Translational Research, College of Human Ecology, Cornell University, Ithaca, New York

## Abstract

**Importance:**

Resident-to-resident aggression in assisted living facilities can result in physical and psychological harm, but its prevalence is unknown.

**Objective:**

To estimate the prevalence of resident-to-resident aggression, including physical, verbal, and sexual, among residents in assisted living facilities.

**Design, Setting, and Participants:**

This study used cross-sectional, observational data from a clinical trial, in which residents of assisted living facilities were monitored for events over a 1-month period. All residents of 14 large facilities randomly selected from 2 geographic locations (N = 1067), except those receiving hospice care (n = 11), were invited to participate; 93 died or moved prior to enrollment. There were 33 family and resident refusals; 930 residents were enrolled. Data were collected between May 30, 2018, and August 11, 2022.

**Main Outcomes and Measures:**

The data are from a clinical trial testing the effectiveness of an intervention to reduce resident-to-resident aggression. In addition, the study was designed to assess prevalence using the Time 1 (baseline) data, using a probability sample of facilities to allow for this analysis. Resident-to-resident aggression was identified using a mixed-method, case-finding strategy involving 6 sources: (1) cognitively capable resident reports regarding 22 possible events, (2) direct care staff report, (3) staff member reports collected from event-reporting forms, (4) research assistant observation of events in real time, (5) facility accident or incident reports, and (6) resident records.

**Results:**

The prevalence of resident-to-resident aggression among the 930 participants (mean [SD] age, 88.0 [7.2] years; 738 women [79.4%]) during the past month was estimated to be 15.2% (141 of 930 residents; 95% CI, 12.1%-18.8%). The most common forms of aggression included verbal (11.2% [104 of 930 residents; 95% CI, 8.8%-14.2%]), physical (41 of 930 residents; 4.4% [95% CI, 3.1%-6.3%]), sexual (0.8% [7 of 930 residents; 95% CI, 0.4%-1.6%]), and other (70 of 930 residents; 7.5% [95% CI, 5.5%-10.2%]). These categories are not mutually exclusive as residents could be involved with more than 1 type of aggressive behavior.

**Conclusions and Relevance:**

In this cross-sectional, observational prevalence study, resident-to-resident aggression in assisted living facilities was highly prevalent. Verbal aggression was the most common form, and physical aggression also occurred frequently. The effects of resident-to-resident aggression can be both morbid and mortal; therefore, intervention research is needed to prevent it and to treat it when it occurs.

## Key Points

**Question:**

What is the prevalence of resident-to-resident aggression (RRA) among residents of assisted living facilities?

**Findings:**

Data from a cross-sectional study using a probability sample of assisted living facilities in New York state showed that the 1-month prevalence of RRA was estimated to be 15.2%. The most common forms of RRA included verbal, physical, and sexual aggression.

**Meaning:**

The relatively high prevalence of RRA among residents of assisted living facilities underscores the need for recognition and treatment to avoid serious consequences for residents.

## Teaser

This cross-sectional study estimates the prevalence of resident-to-resident aggression, including physical, verbal, and sexual, among residents in assisted living facilities.

## Introduction

More than 800 000 individuals in the US live in 30 600 assisted living facilities with a capacity of 1.2 million beds.^1^ Assisted living facilities are settings in which residents typically have better mobility and cognition than in nursing homes, with lower staffing levels. The state survey process is less uniform in assisted living facilities compared with nursing homes. There is no standard definition of *assisted living*, but the term is typically defined as care settings that provide room and at least 2 meals, assistance with personal care, and round-the-clock supervision.^2^

Most assisted living facility residents are non-Hispanic White (89%), female (67%), and older (55%; aged ≥85 years).^3^ Relative to nursing homes, fewer assisted living facility residents are covered by Medicaid (19% vs 60%). Nearly two-thirds of assisted living facility residents require assistance with 3 or more activities of daily living (ADLs), and about two-thirds of residents (66%) have received a diagnosis of at least 2 of the 10 most common chronic conditions among older adults. Alzheimer disease and other dementias are common conditions among assisted living facility residents (42%).^4^ In response to these levels of dementia, assisted living facilities may apply for special certification to provide care to residents with cognitive impairment. The current size and anticipated growth of the assisted living facility sector has led to increased research on the determinants of the quality of care in these settings.

One underresearched area is a problem that clinical experience suggests is widespread: negative and aggressive interactions among residents. Research on nursing homes shows that resident-to-resident aggression (RRA) is highly prevalent. The most extensive study of RRA in nursing homes^5^ found a 1-month prevalence rate of 20.2%. Resident-to-resident aggression has been found to be associated with physical harm and psychological distress and can result in death.^6,7,8,9,10,11^

The fact that assisted living facilities care for individuals who are impaired in activities of daily living, many of whom have dementia, suggests that RRA may also be prevalent in this population. To date, 1 small-scale study has examined the prevalence of RRA among residents in assisted living facilities. In a sample of 121 residents in 6 assisted living facilities, Trompetter and colleagues^10^ found that 19% of the sample reported experiencing what they termed as *resident-to-resident relational aggression*. Given the lack of other research on this potentially prevalent and harmful phenomenon, there is a need to identify the prevalence of RRA in assisted living facilities. This article provides prevalence estimates from the first large-scale, systematic study of RRA in assisted living facilities. Our goal was to estimate RRA prevalence, including subtypes of verbal, physical, and sexual aggression, and to examine variations in the prevalence across demographic and other characteristics.

## Methods

### Design

The design of the prevalence study was cross-sectional (NCT03383289). Estimates of prevalence were obtained using baseline data from an interventional study of RRA. The design of the parent study is described elsewhere.^12^ This study followed the Strengthening the Reporting of Observational Studies in Epidemiology (STROBE) reporting guideline. The study was approved by the Weill Cornell Medicine institutional review board and that of the Hebrew Home at Riverdale. Residents provided written or witnessed oral consent; for residents unable to complete the written or witnessed oral consent process (eg, due to cognitive impairment, language barrier, or health impairment), consent was sought by designated proxies (families or legal guardians).

### Study Population

#### Recruitment of Facilities

Random samples of 6 licensed assisted living residences in New York City, Westchester County, and Long Island (referred to as *downstate*) and 8 licensed assisted living residences in upstate New York were selected, providing downstate and upstate samples, respectively. Both regions were a mixture of urban and suburban. An aim of the study was to examine RRA among all residents, including those with Alzheimer disease. Therefore, to maximize resources, the sample was restricted to larger facilities with special units for residents with Alzheimer disease and other cognitive impairments as reported by the New York state listing of assisted living facilities. In the upstate sample, we selected from the population of 33 facilities with bed sizes of 50 or more with special needs (memory care) units. In the downstate sample, there were 50 larger (≥70 beds) facilities with special needs units.

Of 18 facilities randomly selected, 14 agreed to participate, yielding a participation rate of 77.8%. The relatively high participation rate is in part because these data were collected as part of a clinical trial that offered staff training and in part because of a set of practices that we have used successfully in previous studies, including offering training in RRA intervention to usual care facilities after the data were collected. An honorarium to cover staff time and related costs was provided. In addition, all assisted living communities that we approached perceived RRA as a highly relevant issue to their staff, residents, and families.

#### Eligible Participants

All long-stay residents (N = 1067) except those in hospice (n = 11) were invited to participate. Residents unable to respond were excluded from self-reported measures; however, medical record review, staff informant, and observational measures were performed for individuals with proxy consent. Residents who met the exclusion criteria or who died or were discharged prior to enrollment (n = 93) were excluded from the denominator in prevalence estimates. There were 33 family and resident refusals. The sample size totaled 930 residents (360 upstate and 570 downstate), slightly lower than the preplanned sample of 1050.

#### Procedures

The research team entered each facility for approximately 2 to 3 months and enrolled participants on a rolling basis. A 2-stage cognitive capacity screening test was administered to assess the ability to provide consent for participation in noninvasive research, with a second-stage screening test to assess the ability to provide an extended RRA interview.

Because the protocol was to interview staff first and then residents as soon as possible after the staff interview (usually within 2 weeks), it was necessary to include a 1-month prevalence period to consider reports from both staff and residents. The date of the earliest RRA staff or resident interview (which inquired about events in the prior 2 weeks) bracketed a 1-month period. The staff interview was almost always used to set the index date. For the 57 cases in which only a resident interview was available, that was the index date. Accordingly, event reports, incident reports, and event log data collected during this same 4-week period were selected as potential RRA events by a computer algorithm if they were in the specified date range. Baseline data collection occurred from May 30, 2018, through August 11, 2022.

#### RRA Measures and Case Finding

A triangulation approach for identification of RRA was used; each component contributed to case finding and overall prevalence estimates. Six methods were used to identify cases of RRA over the observation period. An RRA event is defined as any of the following events occurring during the prevalence periods:

##### Resident RRA Instrument

Residents with sufficient cognitive capability were administered an RRA instrument that inquired about 22 forms of physical, verbal, or sexual events in the prior 2 weeks.^13,14^ Physical events included 8 items: hitting, kicking, grabbing, pushing, biting, scratching, spitting, and throwing things; verbal events included 5 items: using bad words, screaming, trying to scare with words, bossing you around, and insulting a resident’s racial or ethnic group; and sexual events included 3 items: saying sexual things, doing sexual things in front of you, and touching in a sexual manner.

##### Staff RRA Instrument

For all consented residents regardless of cognitive status, the primary direct caregiver for the resident was interviewed with the staff version of the instrument.^14^

##### RRA Event Form (Shift Coupon)

Staff completed an RRA event form as events were observed. This preprinted prescription-sized pad with detachable sheets had basic information about events, and completed forms were deposited in a box at the nursing station.

##### Observation or Event Log

A small number of events were directly observed by research staff who were continuously stationed in each facility during the study period and were reported in the event logs. In addition, certain events that were described for one participant in an interview were described for the other participant in event logs.

##### Incident Reports

Facility incident reports were reviewed over the prevalence period for episodes of RRA.

##### Resident Record Reviews

Because in assisted living resident records are inconsistent among facilities, we obtained the state-mandated Assisted Living Residence Medical Evaluation and Resident Evaluation Forms.

#### Covariates

Covariate data were collected to assess whether selected participant, environmental, and facility characteristics were associated with RRA. Respondents were administered The Comprehensive Assessment and Referral Evaluation Dementia Diagnostic Scale assessment.^15,16,17^ This 14-item measure permits 5 classifications of cognitive impairment: none, mild, moderate, severe, and very severe. The Cronbach α coefficient estimated for this sample was 0.87 (ordinal α was 0.94), and the McDonald ω total estimate from a single common factor model was 0.94. The main source of race and ethnicity data was the resident record reviews and, if missing, from the resident interview. The categories were Asian, Black, Hispanic, and White.

In the prior study of RRA prevalence in nursing homes, all RRA events, irrespective of reporting source, underwent a case conference and adjudication process developed for the study.^5^ The purpose of this process was (1) to achieve consensus on cases of RRA that were deemed by 1 or more investigators to be equivocal and (2) to designate a “primary” (ie, most egregious, with the highest risk for harm) form of RRA when multiple types of RRA occurred over the prevalence period. Because it was observed that the agreement was high and the final designation almost always matched what was reported in the resident or staff interview, for this study, case conferencing was not performed.

### Statistical Analysis

The χ^2^ test of significance with SE adjustments for clustering was conducted for comparison of subgroup rates. To estimate prevalence rates, the SPSS Complex Samples Tabulate command in SPSS, version 28.0 (SPSS Inc) was used^18^; 95% CIs were calculated for each estimate. Estimates were adjusted for clustering within units and staff reporters. Estimates from a generalized linear models module^19,20^ were obtained in sensitivity analyses assuming a binomial distribution with a logit link, with inclusion of random effects for unit and staff. Sample size was determined for the clinical trial.

Subtypes of RRA (physical, verbal, and sexual) were similarly calculated with adjustments for clustering. The 1-month and annual prevalences (inclusive of 1 month) were estimated. The rates for subtypes were not mutually exclusive. The rates for verbal subtypes were somewhat higher because some residents engaged in verbal RRA in addition to physical, sexual, or other RRA.

To compare rates by resident characteristics and contextual variables, we estimated rates for the total sample and by downstate and upstate status, sex, age, cognitive status, vision, hearing, ambulation, wheelchair use, unit type, type of room, and season. Based on prior literature on RRA, these factors were considered likely to be associated with RRA prevalence. Rates for downstate and upstate assisted living facilities were not significantly different; thus, the pooled sample was used in subgroup analyses. All statistical tests were 2-sided, and statistical significance was assessed at *P* < .05.

## Results

### Sample

Of the 930 residents (mean [SD] age, 88.0 [7.2] years), 738 [79.4%] were women, and 192 (20.6%) were men (Table 1). Data on race and ethnicity were available for 917 residents: 5 (.05%) were Asian, 11 (1.2%) were Black, 9 (1.0%) were Hispanic, and 892 (97.3%) were White. Most residents were never or no longer married (695 of 844 [82.3%]), and 369 (39.7%) resided in a memory care unit. There were 51 units and 195 staff members. The mean (SD) unit cluster size was 18.2 (12.5) residents per unit. The mean (SD) cluster size for staff was 4.8 (9.5) residents per staff member; the median was 2 (IQR, 1-5). As in previous analyses of this type, the facility variance component was estimated at 0 and was not significant. The variance component from the random-effects model for unit was not significant; the variance component for staff members was significant. The intracluster correlation coefficient was 0.127 for unit and 0.261 for staff. Given the relatively high intracluster correlation coefficient for unit, it was decided to retain both unit and staff as random effects to model the clustering induced by residents clustered within units and within staff members.

**Table 1.** Sample Characteristics

Characteristic	No. (%) (N = 930)
Age, mean (SD), y^a^	88.0 (7.2)
Educational level, mean (SD), y^b^	14.3 (2.9)
Sex	
Female	738 (79.4)
Male	192 (20.6)
Race and ethnicity^c^	
Asian	5 (0.5)
Black	11 (1.2)
Hispanic	9 (1.0)
White	892 (97.3)
Marital status^d^	
Married	149 (17.7)
Widowed	547 (64.8)
Never married	101 (12.0)
Separated or divorced	47 (5.6)
Region	
Upstate New York	360 (38.7)
Downstate New York	570 (61.3)
Residing on a memory care unit	369 (39.7)

^a^ Age available for 926 participants.

^b^ Educational level available for 699 participants.

^c^ Race and ethnicity data available for 917 participants.

^d^ Marital status was available for 844 participants.

### Prevalence Estimates

Prevalence estimates were examined across 2 time periods: 1 month and annual prevalences (inclusive of 1 month) were estimated during the stay at the facility. As shown in Table 2, the 1-month prevalence of RRA was estimated to be 15.2% (141 of 930 residents [95% CI, 12.1%-18.8%]). Most events were verbal (104 of 930 residents; 11.2% [95% CI, 8.8%-14.2%]); however, 4.4% (41 of 930; 95% CI, 3.1%-6.3%) of events involved physical aggression, 0.8% (7 of 930; 95% CI, 0.4%-1.6%) involved sexual aggression, and 7.5% (70 of 930; 95% CI, 5.5%-10.2%) involved other types of aggression (ie, taking and/or damaging a resident’s possessions, threatening gestures, unwanted entry into another resident’s room, forcing unwanted help, unwanted nonsexual touching, and tripping another resident). These were not mutually exclusive categories, and residents could be involved with more than 1 type of aggression. In terms of being involved in RRA during the past year, the estimated rates were higher. Almost one-quarter of residents, (217 of 930; 23.3% [95% CI, 19.6%-27.6%]) were involved annually in any RRA; 18.2% (169 of 930 [95% CI, 15.0%-21.8%]) were involved annually in verbal RRA, 7.4% (69 of 930 [95% CI, 5.4%-10.1%]) were involved annually in physical RRA, 1.1% (10 of 930 [95% CI, 0.6%-2.0%]) were involved annually in sexual RRA, and 13.5% (126 of 930 [95% CI, 10.7%-17.0%]) were involved annually in other RRA. For those involved in RRA during the annual period (n = 217), most events were reported by staff (56.7% [123 of 217]), followed by resident assessment (29.0% [63 of 217]), event logs (28.6% [62 of 217]), shift coupons (13.8% [30 of 217]), and incident or accident reports (5.1% [11 of 217]). A little more than one-quarter of events (27.2% [59 of 217]) were reported by multiple sources.

**Table 2.** Prevalence Estimates of RRA Across Various Observation Periods (N = 930)

RRA occurrence and type	Adjusted value, No. (%) [95% CI]^a^
1-mo Time period	
Any RRA incident	141 (15.2) [12.1-18.8]
Verbal	104 (11.2) [8.8-14.2]
Physical	41 (4.4) [3.1-6.3]
Sexual	7 (0.8) [0.4-1.6]
Other	70 (7.5) [5.5-10.2]
Annual rate	
Any RRA incident	217 (23.3) [19.6-27.6]
Verbal	169 (18.2) [15.0-21.8]
Physical	69 (7.4) [5.4-10.1]
Sexual	10 (1.1) [0.6-2.0]
Other	126 (13.5) [10.7-17.0]

Abbreviation: RRA, resident-to-resident aggression.

^a^ Values are adjusted for clustering within staff member and unit.

Estimates by region and residence in memory care units are presented in Table 3. Prevalence estimates during the 1-month period ranged from 3.2% (3 of 95) to 26.4% (14 of 53) in downstate facilities and 3.6% (1 of 28) to 38.5% (20 of 52) in upstate facilities. Annual prevalence estimates ranged from 8.4% (8 of 95) to 36.1% (30 of 83) in downstate and 5.6% (1 of 18) to 50.0% (26 of 52) in upstate facilities. Three upstate facilities had estimated rates over 30.0% (31.7% [19 of 60], 42.4% [28 of 66], and 45.0% [18 of 40]). Twice as many residents in memory care units were reported to engage in RRA compared with individuals residing in other units (22.5% [83 of 369] vs 10.3% [58 of 561]).

**Table 3.** One-Month Prevalence Estimates by Resident, Region, Facility, and Unit Characteristics

Characteristic	Adjusted value	
	No.	% (95% CI)^a^
Sex		
Male	192	13.5 (9.1-19.7)
Female	738	15.6 (12.2-19.7)
Age categorized, y		
<75	45	20.0 (10.0-36.1)
75-84	216	17.6 (12.6-24.0)
≥85	665	14.1 (11.1-17.8)
Age dichotomized, y		
<85	261	18.0 (13.0-24.4)
≥85	665	14.1 (11.1-17.8)
Categorized CARE diagnostic scale classifications		
None (0-3)	105	19.0 (11.9-29.1)
Mild (4-6)	84	15.5 (9.0-25.3)
Moderate (7-10)	140	20.7 (13.6-30.2)
Severe (11-15)	68	17.6 (9.9-29.4)
Very severe impairment with communication problem (nontestable; ≥16)	56	25.0 (14.6-39.5)
Collapsed CARE diagnostic categories		
None or mild	189	17.5 (11.8-25.0)
Moderate cognitive impairment	140	20.7 (13.6-30.2)
Severe or very severe cognitive impairment	124	21.0 (14.1-30.0)
Vision^b^		
No impairment or slight impairment	413	21.1 (16.5-26.5)
Moderate or severe impairment or blind	85	7.1 (2.8-16.8)
Vision could not be assessed	297	13.5 (9.8-18.2)
Hearing^b^		
No impairment or slight impairment	585	18.6 (14.8-23.1)
Moderate or severe impairment or deaf	119	12.6 (7.7-20.0)
Hearing could not be assessed	97	10.3 (5.3-19.2)
In wheelchair during interview		
No	429	19.3 (14.5-25.3)
Yes	41	14.6 (7.0-28.1)
Ambulation status^b^		
Not able to ambulate	198	10.6 (6.9-16.0)
Can ambulate	616	18.5 (14.7-23.0)
Region, facility, or unit		
Region		
Upstate	360	19.7 (14.3-26.6)
Downstate	570	12.3 (9.1-16.4)
Memory unit vs other unit^b^		
No	561	10.3 (7.5-14.0)
Yes	369	22.5 (17.2-28.9)
Type of room		
Private	806	16.0 (12.7-20.0)
Shared	69	13.0 (6.9-23.2)
Season interview took place^c^		
Winter (December 21 to March 19)	79	11.4 (4.5-26.0)
Spring (March 20 to June 19)	166	13.3 (8.1-21.0)
Summer (June 20 to September 22)	561	16.4 (12.5-21.3)
Fall (September 23 to December 20)	123	14.6 (8.4-24.3)

Abbreviation: CARE, Comprehensive Assessment and Referral Evaluation.

^a^ Percentages are nonduplicated cases and are adjusted for clustering within staff member and unit. Percentages are in reference to each category.

^b^ Significant comparisons based on the χ^2^ test with adjustment for clustering as follows: no visual impairment or slight visual impairment vs moderate or severe visual impairment or blind (*P* < .001); no hearing impairment or slight hearing impairment vs moderate or severe hearing impairment or deaf (*P* = .007); ambulation status (*P* = .005); and residing on memory care unit (*P* < .001).

^c^ The interview season is a proxy for the event season for the 1-month lookback period.

Table 3 also shows prevalence rates by selected demographic, functional, and cognitive characteristics. As shown, rates were not significantly different across groups differing on sex, age, and cognitive status. For example, 18.0% of those younger than 85 years of age (47 of 261) vs 14.1% of those 85 years or older (94 of 665) engaged in RRA; however, the largest proportion (20.0% [9 of 45]) was observed in the group younger than 75 years.

Significantly more residents with normal vision or slight vision impairment engaged in RRA, in contrast with those with moderate to severe vision impairment (21.1% [87 of 413] vs 7.1% [6 of 85]); the same was found for residents with normal hearing or slight hearing impairment vs those with moderate to severe hearing impairment (18.6% [109 of 585 vs 12.6% [15 of 119]) (Table 3). Ambulation ability was also associated significantly with RRA (18.5% [114 of 616] vs 10.6% [21 of 198] among those who did not ambulate).

## Discussion

It was expected that lower rates of RRA would be observed among residents of assisted living facilities than in a previous study of nursing homes,^5^ because of a lower prevalence of cognitive impairment and the greater privacy and space afforded to residents of assisted living facilities. However, although lower, the prevalence was higher than expected, with a 1-month estimate of 15.2%. The annual rate was observed to be almost one-quarter (23.3%) of residents. In the nursing home study, the comparable 1-month and annual rates were 20.2% and 25.2%, respectively,^5^ which are not substantially higher than those observed in assisted living facilities. Consistent with prior RRA studies, verbal RRA was the most common in our study sample.

Although there were few differences observed in rates across subgroups, the prevalence of RRA in memory care units was significantly higher than other units (22.5% vs 10.3%). This finding aligns with findings from a prior nursing home study using a nearly identical methodological approach, with rates of 29.0% in dementia care units vs 18.5% in nondementia care units.^5,21^ Potentially associated with this finding is that a larger proportion of residents (39.7%) resided in memory care units in our assisted living facility sample as contrasted with 16.3% in the nursing home sample.^5,21^

Other research has also found higher prevalence estimates of RRA among residents in memory care units. An earlier retrospective case record study has shown that the incidence of aggressive behavior per resident per year was almost 3 times as likely among residents in memory care units than among residents in the rest of the facility.^21^ A major reason for this higher prevalence is that individuals with dementia and behavior problems resulting from it are congregated closely. The environment is therefore characterized by greater opportunities for contact with other residents who are disinhibited and thus at higher risk for aggressive interactions.^22^ As such, targeting increased RRA recognition and prevention training to memory care unit staff may result in significant reductions of its occurrence.

Other comparable findings between the present study and prior nursing home research^5^ pertain to residents’ functioning in the areas of physical mobility and vision. A significantly greater prevalence of RRA was observed among residents in both settings who could ambulate (26.4% in the nursing home study and 18.5% in the current assisted living facility study) than among those who could not (11.9% in the nursing home study and 10.6% in the current assisted living facility study). In addition, both settings were also characterized by estimates of more RRA among residents who had unimpaired vision as contrasted with those with vision impairment. This difference was more pronounced in assisted living facilities. Similarly, in assisted living facilities, the prevalence of RRA was significantly lower among those with moderate to severe hearing impairment (12.6%) than those with normal hearing or slight hearing impairment (18.6%), perhaps because they could not engage as easily in verbal altercations.

Several areas of divergence exist between our present study and findings in the nursing home study.^5^ One difference is in the level of cognitive impairment and the prevalence of RRA. In prior RRA nursing home literature,^5^ individuals with less cognitive impairment were more likely to be involved in RRA. Although it might be expected that individuals with severe cognitive impairment may be more at risk, such was not the case in nursing homes. This finding was explained by the fact that persons with earlier stages of dementia are more mobile and thus have more opportunities to engage in aggressive behaviors with other residents. In contrast, individuals in nursing homes with very severe cognitive impairment were often at the end stages of dementia with limited mobility and were unable to engage in such behaviors.^15,16^ In contrast, in the assisted living facility sample, the rates across cognitive categories were more similar. This finding lends support to furthering a more nuanced understanding of cognitive function in association with RRA occurrence across diverse long-term care settings.

### Limitations

This study has several limitations that point to directions for future research. Data were collected in part during the COVID-19 pandemic, and staffing shortages and temporary closures resulted in some missing data. For example, there were 57 cases with only resident-reported data rather than both resident-reported and staff-reported data. This pattern may have resulted in underreporting of RRA among those residents. Because of deaths in the assisted living facilities, the expected sample size was smaller (n = 930) than planned (n = 1050); the association of this difference was primarily seen in subgroup comparisons, which were based on reduced group sizes. It was also not possible to perfectly line up the staff and resident interviews within a 2-week period; thus, 1 month was used as the reference. In terms of generalization, there were similarities and differences from national averages.^1^ This sample consisted of larger facilities, 50 beds or more, which represents the minority of facilities nationally. The sample was of assisted living facilities with special needs units; only 29% of licensed facilities nationally provide such care. The sample included 79.4% women, similar to about 70% nationally, and the mean (SD) age was 88.0 (7.2) years, consistent with a median age of 85 years nationally. Although the residents were predominantly White, this sample mirrors the national composition of residents of assisted living facilities.^1^ Future research would benefit from oversampling residents of racial and ethnic minority populations to better understand whether known racial and ethnic disparities in long-term care may extend to RRA in the assisted living facility landscape.

## Conclusions

Despite these limitations, to our knowledge, these are the only data available from a large sample of well-characterized residents of assisted living facilities. These data show that the prevalence of RRA in assisted living facilities was high. It was expected that the larger space afforded residents in assisted living facilities, coupled with primarily private rather than shared rooms, would reduce the circumstances in which RRA might be observed. However, a partial explanation for these rates may be the relatively large proportion (39.7%) of residents in assisted living memory care units who experience higher levels of RRA.

Taken together, our findings underscore the need to accelerate the development and implementation of RRA interventions. Such efforts should include tailoring and adapting an existing evidenced-based RRA intervention for use in other congregate care settings.^23^ Policymakers should also consider reviewing and expanding current regulations and protocols to include policies for preventing, reporting, and treating RRA to ensure the safety of the increasing number of assisted living facility residents.

## Notes

Supplement.
Data Sharing Statement
