# Supplementary material for: Estimated Prevalence of Resident-to-Resident Aggression in Assisted Living
Source: JAMA Netw Open. 2024 May 3;7(5):e249668. doi: 10.1001/jamanetworkopen.2024.9668 (PMC11069077; doi:10.1001/jamanetworkopen.2024.9668)
Supplement: Supplement. — Data Sharing Statement [file jamanetwopen-e249668-s001.pdf]

Data Sharing Statement

Pillemer. Estimated Prevalence of Resident-to-Resident Aggression in Assisted Living. *JAMA Netw Open*. Published May 03, 2024. doi:10.1001/jamanetworkopen.2024.9668

Data
Data available: Yes
Data types: Data dictionary
How to access data: Data requests can be made to Joseph P. Eimecke, jpeimicke@aol.com
When available: With publication

Supporting Documents
Document types: None

Additional Information
Who can access the data: Researchers whose proposed use of the data has been approved
Types of analyses: For research purposes
Mechanisms of data availability: After approval of a proposal
